# A Comparative Analysis of the Clinical and Radiological Results of a Zero-Profile Device Versus Conventional Cage and Plate Following Single-Level Anterior Cervical Discectomy and Fusion

**DOI:** 10.7759/cureus.80067

**Published:** 2025-03-04

**Authors:** Sumira Kiran, Zubair M Khan, Khawar Anwar, Haseeb Mehmood Qadri, Sundas Irshad, Ch. Arslan Ahmad, Raahim A Bashir, Manal Khan, Maksalmina Reshtin, Asif Bashir

**Affiliations:** 1 Neurological Surgery, Punjab Institute of Neurosciences, Lahore, PAK; 2 General Surgery, Lahore General Hospital, Lahore, PAK

**Keywords:** anterior cervical discectomy and fusion, dysphagia, pakistan, prevertebral soft tissue thickness, zero-profile

## Abstract

Background

Conventional cage and plating (CCP) and stand-alone self-interlocking zero profile cage (ZPC) are the two devices used in anterior cervical discectomy and fusion (ACDF) for the treatment of cervical spondylosis refractory to medical treatment. The utilization of zero-profile implants in ACDF for the management of degenerative cervical spondylosis has gained popularity. Nevertheless, the available evidence regarding its effectiveness and safety remains insufficient.

Objective

To compare the clinical and radiological outcomes of CCP and ZPC for single-level ACDF.

Methodology

In this retrospective cohort study, the records of patients who underwent single-level ACDF with CCP and ZPC between December 2021 and December 2023 were recruited in December 2024. Patients with ossified posterior longitudinal ligament, history of previous cervical surgery, severe comorbidities, older than 70 years and patients who utilized the circumferential fusion approach in combination were excluded from the study. The means and frequencies of clinical and radiological outcomes were compared between two groups via Mann-Whitney test, Fisher’s exact test, and Chi-square test of significance, where p<0.05 was considered significant. The six-month follow-up data were recorded for all the patients included in the study.

Results

Of the 53 included patients, CCP was utilized in 30 (56.6%) and ZPC in 23 (43.4%) patients. The mean age of study participants was 53.8±9.3 years, with 35 (66%) males and 18 (34%) females. The ZPC implant resulted in reduced blood loss (p=0.001). However, no statistically significant difference was observed in operation time and the incidence of postoperative transient dysphagia between the two groups (p=0.532, p=0.569, respectively). Additionally, the ZPC implant demonstrated a significantly lower occurrence of postoperative dysphagia at two weeks and six months postoperatively (p=0.015 and p=0.039 respectively) compared to the CCP implant.

Conclusion

The utilization of ZPC may lead to positive clinical and radiological outcomes and decrease the frequency of postoperative dysphagia in ACDF procedures involving a single level.

## Introduction

Cervical spondylosis is one of the most common natural aging pathology with a prevalence of 75% when the person reaches the age of 65 [1]. Initial management is focused on conservative treatment with pain medications, muscle relaxants, and physiotherapy. If the symptoms progress, surgical treatment is considered [2]. The most common surgical modality offered to the patients is anterior cervical discectomy and fusion (ACDF) [3]. ACDF was first introduced by Smith and Robinsons in 1958 and later evolved by Cloward [4]. Anterior Cervical discectomy focuses on the decompression of spinal nerve roots and spinal cord, along with restoration of spinal curvature and fusion of adjacent vertebrae [5]. Since the inception of ACDF techniques, different materials have been used for this procedure, starting from bone graft to conventional cage and plate (CCP) [6]. Recently, much interest has been generated in using the zero profile cage (ZPC) [7,8]. As compared to CCP, ZPC has been shown to have less operative time, less damage to the neurovascular structure, and fewer tractional injuries [9]. Additionally, it has been shown to have a higher level of cage subsidence with loss of disc height and disruption of sagittal alignment of the spine [10].

The purpose of this study was to assess the clinical and radiological outcomes of the ACDF in cervical spondylotic disc disease. We report our early experience with conventional single-level cage (CCP) vs Stand-alone self-interlocking zero profile cage (ZPC), followed up to six months after surgery.

## Materials and methods

This retrospective cohort study was conducted at the Department of Neurosurgery, Punjab Institute of Neurosciences, Lahore, Pakistan in December 2024 involving 53 patients who received ACDF treatment from December 2021 to December 2023, comparing the clinical and radiological outcomes of CCP and ZPC in ACDF.

Inclusion criteria

All the included patients had typical symptoms of cervical radiculopathy or myelopathy (posterior neck pain, radiating pain, and limb numbness), MRI findings of spinal cord ventral compression mainly caused by single-level cervical disk herniation and unsatisfactory results with medical treatment tried for at least two months.

Exclusion criteria

We excluded the patients with ossification of posterior longitudinal ligament, patients with severe co-morbidities, patients having previous cervical surgery for any reason, patients older than 70 years, and patients who utilized the circumferential fusion approach in combination.

Data collection

The records of included patients were reviewed, which revealed 30 patients in the CCP group and 23 patients in the ZPC group. The data regarding the demography of patients, surgical technique employed, and clinical and radiological outcomes of patients was gathered in a self-designed questionnaire. The majority of patients included in our study were followed up for six months after the study, due to which we focused mainly on short-term outcomes.

Surgical technique

In both groups, the standard anterior Smith-Robinson approach [11] was employed after supine positioning of patients with a slightly extended head. After dissection of skin, subcutaneous tissue and platysma, the sternocleidomastoid muscle was retracted laterally followed by palpation of the deep neck structures. In ZPC group, once the operation levels were confirmed, a Cloward distractor blade was positioned under the longus colli muscle. Disc removal was done using a Kerrison punch and pneumatic drill. Following the use of trial spacers to ensure the correct polyetheretherketone (PEEK) spacer, an allograft-filled ZPC implant was installed at the level of operation. Radiological imaging in anteroposterior and lateral planes via fluoroscopy was utilized to determine the optimal point for accurate positioning of the ZPC spacer. An aiming device was used to drill the first pilot hole after which the first locking screw was secured by using a torque limited to 1.2 Nm. Similarly, rest of the locking screws were fixed while creating a bone wedge at various angles in craniocaudal and mediolateral dimensions i.e., 40±5° and 2.5° respectively while keeping the standard diameter of 3 mm. Although various lengths ranging from 12 to 16 mm were evaluated, we found 16 mm as the most suitable length for the majority of patients. Alternatively, in the CCP group, a PEEK cage combined with a Zephir anterior cervical plate was utilized. The Zephir plate had a width of 15 mm and a thickness of approximately 1.6 mm. Its length started from 22.5 mm with a maximum length of 70 mm. The appropriate length was decided individually for each case. All the surgeries were performed by two primary surgeons who did surgery together.

Clinical and radiological outcomes

The modified Japanese Orthopedic Score (mJOA) was used to assess clinical outcomes [12]. These outcomes were evaluated both before the surgery, at the time of admission, and six months after the surgery. The mJOA score achieved was calculated by subtracting the preoperative mJOA score from the postoperative mJOA score. Additionally, the recovery rate (RR) was determined as a percentage using the formula [(postoperative mJOA score - preoperative mJOA score)/(18 - preoperative mJOA score)] × 1006 [13]. The time of operation and estimated blood loss (EBL) were also estimated.

Similarly, all the included patients were regularly reviewed on the day of admission or during the pre-operative evaluation period for the cervical spine surgery, utilizing multiple views (oblique view, anteroposterior view, lateral view, excessive flexion, and extension views) on plain radiological films of the cervical spine, along with MRI and computed tomography (CT) scans for the assessment of radiological findings. Follow-up appointments were scheduled for a maximum period of six months on an outpatient basis, during which radiological imaging (plain x-ray films, CT scans, and MRIs) were conducted. The evaluation of prevertebral soft tissue thickness (PSTT) was carried out before surgery, on the day of the surgery, as well as after two weeks of surgery and after six months of surgery. By utilizing the lateral views from the cervical plain film, the PSTT was calculated as the average distance between the air shadow of the airway and the anterior margin of each vertebral body or plate. These measurements were then compared to the data collected during pre-operative evaluation to determine the differences between the two groups. The analysis of radiological imaging was conducted, independently, three times to ensure precision.

Data analysis

Statistical analyses were conducted utilizing Statistical Package for Social Sciences Software (IBM SPSS Statistics For Windows, version 24, released 2016; IBM Corp, Armonk, New York, United States). The data is presented as mean±standard deviation. Quantitative analyses were carried out using the Mann-Whitney test, while for qualitative analyses, we performed Fisher's exact test and Chi-square test. A p<0.05 was deemed to be statistically significant.

## Results

A total of 53 patients were included in this study, of which, there were 35(66%) males and 18(34%) females. The mean age of study participants was 53.8±9.3 years. The majority of the included patients in this study had typical symptomatology of cervical spondylosis including pain in the posterior neck radiating to limbs associated with numbness and paresthesia. The study participants were categorized into two groups based on devices used, i.e., CCP group and ZPC group with 30 and 23 patients respectively. Patients were followed up post-operatively for six months. The demographic and baseline characteristics of study participants are summarized in Table 1 below.

**Table 1:** Baseline characteristics of study participants CCP: conventional cage and plating; ZPC: zero profile cage: M: mean; SD: standard deviation.

Variables	CCP	ZPC	p-value
Gender, n (%)			
Males	22 (73.3)	13 (56.5)	0.249
Females	8 (26.6)	10 (43.4)	
Age (Years), M±SD	51.2±10.3	52.6±9.6	0.153
Operative levels, n (%)			
C3-C4	3 (10)	3 (13)	0.982
C4-C5	7 (23.3)	5 (21.7)	
C5-C6	14 (46.6)	10 (43.4)	
C6-C7	6 (20)	5 (21.7)	
Total, N (%)	30 (56.6)	23 (43.3)	-

The average estimated blood loss (EBL) in the CCP group was 140.6±125.0 ml while the mean EBL in the ZPC group was 89±141.0 ml Similarly, the ZPC group had a lesser mean operation time in comparison to the CCP group, i.e., 143±49.6 min versus 156.4±53.2 min, respectively. The preoperative and post-operative modified JOA (mJOA) score, Achieved mJOA score and Recovery rate of mJOA score of both groups along with their level of significance are described below in Table 2. The results showed a significant mean difference in EBL between both groups with a p-value of 0.031.

**Table 2:** Comparison of clinical outcomes among CCP group vs ZPC group CCP: conventional cage and plating; ZPC: zero profile cage; JOA: Japanese Orthopedic Score; M: mean; SD: standard deviation; *p<0.05: significant.

Variable	CCP	ZPC	p-value
Estimated Blood loss( ml), M±SD	140.6±125.0	89±141.0	0.031*
Operation Time (min), M±SD	156.9±53.2	143±49.6	0.043*
Preoperative modified JOA score, M±SD	15.5±1.3	15.9±1.4	0.560
Postoperative modified JOA score, M±SD	17.1±0.9	17.7±0.9	0.431
Achieved modified JOA score, M±SD	1.6±0.9	1.8±0.9	0.215
Recovery rate of modified JOA score, (%)	63.0±19.0	85.2±20.1	0.453

Regarding radiological outcomes, the prevertebral soft tissue thickness of patients calculated pre-operatively, early post-operatively, two weeks after surgery, and six months after surgery, of both CCP and ZPC groups are described below in Table 3. All the values of PSTT among both groups were statistically similar except the values calculated after six months postoperatively with p=0.005.

**Table 3:** Comparison of radiological outcomes (PSTT, mm) CCP: conventional cage and plating; ZPC: zero profile cage; PSTT: prevertebral soft tissue thickness; M: mean; SD: standard deviation; *p<0.05 significant

Variable	CCP	ZPC	p-value
Preoperative (mm), M±SD	12.1±0.8	13.4±1.6	0.398
Operative day, M±SD	15.4±2.3	15.0±2.4	0.436
Postoperative (2 weeks), M±SD	17.1±2.4	15.6±2.2	0.305
Postoperative (6 months), M±SD	15.1±1.9	14.5±2.7	0.005*

The incidence of postoperative dysphagia was calculated as a complication of ACDF on the day of surgery, after two weeks of surgery, and after six months of surgery. The incidence of postoperative dysphagia in both groups along with the level of significance is given below in Table 4.

**Table 4:** Calculated differences in the incidence of postoperative dysphagia among CCP group vs ZPC group *p<0.05 significant

Duration	CCP	ZPC	p-value
Operative day, n(%)	17 (56.6)	12 (52.1)	0.569
Postoperative (2 weeks), n(%)	12 (40)	2 (8.6)	0.015*
Postoperative (6 months), n(%)	7 (23.3)	0 (0)	0.039*

## Discussion

Anterior cervical decompression and fusion (ACDF) is the most frequently performed procedure for the treatment of cervical spondylosis refractory to medical management [14]. With the advancements in the field of neurosurgery, multiple surgical devices employing different surgical techniques have been introduced including conventional cage and plate (CCP) and zero-profile stand-alone interbody spacer (ZPC) devices [15]. Although the rate of fusion, as well as biomechanical stability, is similar in both ZPC and CCP implants, the rate of dysphagia is comparatively lesser with ZPC implants. Moreover, a higher subsidence rate was observed with the ZPC group which leads to reoperation [16]. This study aimed to compare the clinical and radiological outcomes of CCP and ZPC devices while specifically identifying the difference in the incidence of postoperative dysphagia.

The results of our study show a significantly lesser mean operation time, estimated blood loss, and postoperative dysphagia. This is similar to the findings of a study by Noordhoek et al., [17] which shows a significant decrease in intraoperative blood loss, mean operative time, and rate of postoperative dysphagia in patients undergoing ACDF with ZPC implant whereas a non-significant increase in cage subsidence rate was also observed in patients with ZPC implants (p=0.5). Similarly, a significantly higher JOA score was observed in the ZPC group in comparison to the CCP group with a mean difference of  0.17 (p=0.02) [16] which is also evident through the results of our study.

Chronic dysphagia is the most frequently reported complication of ACDF with an incidence rate ranging from 3 to 21% [18]. The presumed causes of post-operative dysphagia after ACDF according to existent literature include hematoma development at the site of surgery, development of post-operative soft tissue swelling, direct injury to the esophagus, and formation of adhesions around the implanted cervical plates [19]. According to Shao et al. [20], the width of anterior plates at the level of fusion determines the occurrence of dysphagia postoperatively in such patients. The volume of the ZPC device is less than that of CCP, hence implying the need for small incision, limited resection, reduced tissue exposure and decreased mechanical disturbance at the site of operation with comparatively lesser damage to surrounding structures, hence affecting the prevertebral soft tissue thickness minimally [20]. We, therefore, hypothesized that the thickness of prevertebral soft tissue would be lesser in the ZPC group in comparison to the CCP group. The comparison of radiological outcomes in this study depicted a clearly significant difference in prevertebral soft tissue thickness among the two groups with the ZPC group showing mild swelling of prevertebral soft tissue post-operatively (p<0.001). The outcomes of a meta-analysis showed a significantly decreased risk of short-term, medium-term, and long-term risk of dysphagia in the ZPC group with an odds ratio of 0.40, 0.31, and 0.29 respectively (p<0.001) [16].

ZPC implant is inserted into the intervertebral disc space without the use of a titanium plate which may reduce the mean operative time in comparison to the CCP group while it is reported in literature that ZPC implant fails to maintain cervical curvature and effective intervertebral height in comparison to CCP group that may lead to increased rate of cage subsidence in ZPC group [21]. Similarly, Li et al., [22] reported lower maximum stress and range of motion in the ZPC group in comparison to the CCP group. This is in contrast to the findings of Kahaer et al. [16], who reported no significant difference in prevertebral soft tissue thickness and cage subsidence rate, correction of cervical curvature, rate of fusion and failure of implant between ZPC and CCP group.

Limitations

This study has several limitations that necessitate additional research in the future. To validate our hypothesis, prospective studies with larger sample sizes and a longer follow-up period are required. Additionally, analyzing multiple levels of operation would provide a more comprehensive understanding of the benefits of the implant. Another limitation is the variation in prevertebral structures between cervical vertebrae at various levels of the spine. The upper cervical level only includes the posterior hypopharyngeal wall in the prevertebral structures, while the lower cervical level has more complex components such as the esophagus, transverse arytenoid muscle, larynx, and trachea. These differences may impact the accuracy of measurements. A more thorough analysis of the association between components of every operative level and swelling of corresponding soft tissue is needed for conclusive results. Lastly, although there was a significant difference in estimated blood loss (EBL) between the groups, the large standard deviation of EBL may have influenced this result due to variations in how EBL was recorded by different anesthesiologists.

## Conclusions

ACDF has been proven to be a reliable procedure for treating diseases related to cervical intervertebral discs. This study found that both CCP and ZPC devices were successful in ACDF done for single-level affected cervical disc, but ZPC spacer leads to a comparatively minimal swelling of the prevertebral soft tissue and reduced incidence of dysphagia at six months postoperatively. To further solidify these findings, it would be beneficial to conduct future studies with a large sample size, prospective approach and a long-term follow-up postoperatively.
